# A Rare Case of Antinuclear Antibody-Negative Systemic Lupus Erythematosus Presenting With Generalized Lymphadenopathy as the Initial Manifestation

**DOI:** 10.7759/cureus.94661

**Published:** 2025-10-15

**Authors:** Nazifa Ibnath, Rajat Biswas

**Affiliations:** 1 Internal Medicine, Chattrogram Maa-O-Shishu Hospital Medical College, Chattogram, BGD; 2 General Medicine, University of Washington Medical Center, Seattle, USA; 3 Medicine, Chattrogram Maa-O-Shishu Hospital Medical College, Chattogram, BGD

**Keywords:** anti-dsdna, corticosteroid therapy, generalized lymphadenopathy, immunologic testing, negative anti-nuclear antibody, pediatric sle, systemic lupus erythematosus, thrombocytopenic purpura

## Abstract

Systemic lupus erythematosus (SLE) is a chronic autoimmune disorder characterized by multisystem involvement and the presence of antinuclear antibodies (ANA). However, a subset of patients, particularly in the pediatric population, present with ANA-negative SLE, posing significant diagnostic challenges. This phenomenon is notably rare in South Asia, where ANA positivity is typically prevalent in SLE cases. The rarity of ANA-negative pediatric SLE underscores the necessity for heightened clinical vigilance and comprehensive diagnostic evaluation.

We report the case of a 16-year-old South Asian female patient who presented with generalized lymphadenopathy (LAD), fever, alopecia, oral ulcers, purpuric rashes, and polyarthritis. Despite the absence of detectable ANA (tested by enzyme-linked immunosorbent assay (ELISA); immunofluorescence (IF) was not performed due to resource limitations), the patient exhibited strongly positive anti-double-stranded DNA antibody (anti-dsDNA) antibodies, fulfilling four American College of Rheumatology (ACR) classification criteria for SLE. The patient responded positively to corticosteroid and hydroxychloroquine therapy, with subsequent resolution of symptoms and stabilization of blood counts.

ANA-negative pediatric SLE is an uncommon but clinically significant entity in South Asia. This case emphasizes the importance of applying comprehensive diagnostic criteria, such as the Systemic Lupus International Collaborating Clinics (SLICC) 2012, when ANA is negative. Clinicians should maintain a high index of suspicion for SLE in pediatric patients with multisystem involvement, ensuring early recognition and treatment to optimize outcomes.

## Introduction

Systemic lupus erythematosus (SLE) is a chronic, multisystem autoimmune disorder with a highly heterogeneous clinical spectrum, defined by the production of autoantibodies, most prominently antinuclear antibodies (ANA) [1-5]. While SLE predominantly affects females, with peak incidence in early adulthood, pediatric-onset cases comprise a smaller yet clinically significant subset [1-4,6]. Early recognition of atypical manifestations is crucial, as delayed diagnosis may precipitate severe organ involvement and heightened morbidity [3,4,6].

SLE results from dysregulation of the immune system, leading to the production of autoantibodies that target self-antigens. These autoantibodies form immune complexes, which deposit in various organs and tissues, triggering complement activation, inflammation, and tissue damage. This immune-mediated pathology underlies the heterogeneous clinical manifestations of SLE, including cytopenias, nephritis, arthritis, and cutaneous lesions.

SLE commonly presents with malar rash, photosensitivity, oral ulcers, nephritis, serositis, hematologic abnormalities, and arthralgia. ANA positivity, detected via immunofluorescence (IF; gold standard) or enzyme-linked immunosorbent assay (ELISA), is traditionally considered a hallmark, present in approximately 95%-99% of patients [5,6]. Yet, a rare subset of patients demonstrates ANA-negative SLE, reported in 2%-5% of cases, presenting unique diagnostic challenges [7-13]. This phenotype is especially uncommon in pediatric populations, rendering each documented case a valuable contribution to the literature [7-13]. Patients with ANA-negative SLE may still exhibit other serologic abnormalities, such as anti-double-stranded DNA or anti-Ro/SSA antibodies, and can present with cytopenias, nephritis, or other systemic manifestations [11-13]. Additionally, even in ANA-positive cases, atypical presentations such as acute pancreatitis have been reported, highlighting the wide spectrum of unusual manifestations that may occur in SLE patients [14].

Generalized lymphadenopathy (LAD) is an uncommon initial manifestation of SLE in current clinical practice. While older studies reported LAD in up to 50%-70% of patients, more recent cohorts suggest a lower prevalence, with figures varying across different studies [2,3,9,10]. Persistent or diffuse LAD frequently prompts consideration of malignancies, infections like tuberculosis, or other lymphoproliferative disorders, often delaying the correct diagnosis of SLE. 

In routine clinical practice, indirect IF (IIF) is considered the gold standard for ANA detection. However, in resource-limited settings such as many centers in South Asia, ANA testing is often performed using ELISA-based methods, alongside anti-double-stranded DNA (anti-dsDNA) assays, which are more widely available and cost-effective. This reliance on ELISA testing may partially account for cases of apparent ANA negativity in patients who otherwise meet clinical and serologic criteria for SLE.

The rarity of ANA-negative SLE manifesting as generalized LAD, particularly in pediatric patients, underscores the need for heightened clinical vigilance. For reference, a full summary of the SLE classification criteria, including American College of Rheumatology (ACR) [15] and Systemic Lupus International Collaborating Clinics (SLICC) 2012 criteria [16], is provided in Appendix A. Here, we present a case of a 16-year-old female patient with ANA-negative SLE whose first manifestation was diffuse, painless LAD, emphasizing that a high index of suspicion must be maintained even when ANA results are negative. 

## Case presentation

A 16-year-old Asian female student presented to the medicine outpatient clinic with a four-month history of progressive, painless lymph node enlargement. The swelling initially developed behind the right ear in the post-auricular region and in the posterior cervical area. Despite a 21-day course of unspecified treatment prescribed at an ear, nose, and throat clinic, the lymph nodes continued to enlarge in both size and number. Over the following months, additional lymph nodes became apparent in the anterior cervical, supraclavicular, axillary, and inguinal regions. During this period, the patient also reported hair loss localized to the frontal scalp, which was gradual and non-scarring.

**Figure 1:**
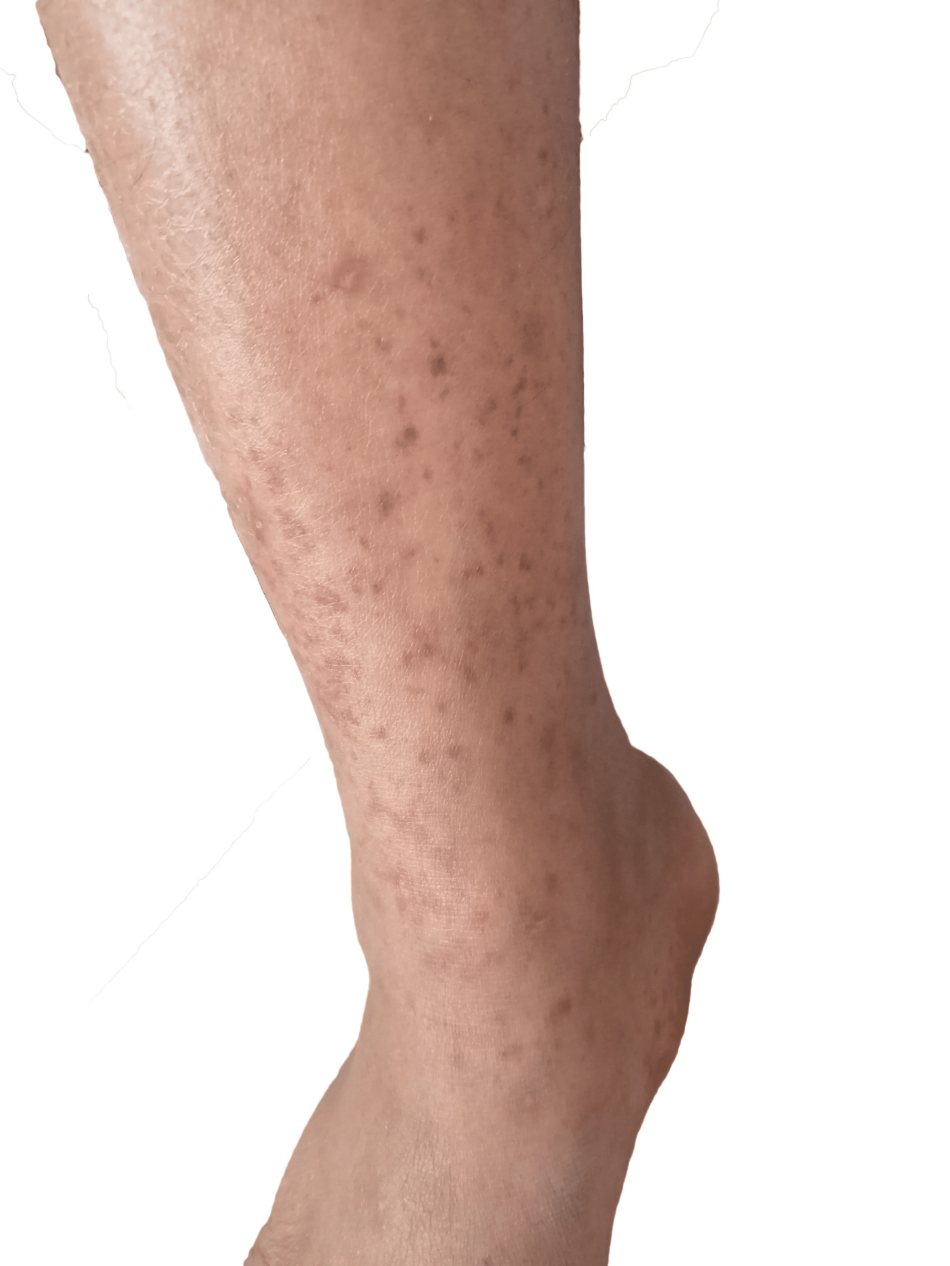
Purpuric lesions on the lower extremities Multiple non-palpable, non-blanching violaceous spots are visible, primarily confined to regions below the buttocks. The lesions appeared shortly after the onset of an intermittent febrile illness. The exact etiology of the rash is uncertain, but it is consistent with cutaneous bleeding and may be related to mild thrombocytopenia or an immune-mediated process. No ulceration, inflammation, or excoriation is observed.

Approximately three months after the onset of LAD, the patient developed intermittent polyarthralgia that evolved into migratory inflammatory joint pain involving the wrists, metacarpophalangeal joints, elbows, knees, and ankles. This was accompanied by mild swelling, warmth, and tenderness over the affected joints. During the same period, she experienced two brief episodes of transient, non-pruritic erythematous rash on the trunk and extremities, each lasting two to three days, and a single painless oral ulcer that healed spontaneously. About fifteen days prior to presentation, she developed an intermittent febrile illness characterized by evening-predominant temperature spikes reaching approximately 38°C, associated with chills. Shortly after the onset of fever, she noticed the development of a purpuric, non-blanching rash confined to the lower extremities below the buttocks, as depicted in Figure 1. She denied photosensitivity, malar rash, mucosal bleeding, hematuria, respiratory or cardiac symptoms, weight loss, night sweats, or any recent significant travel or tuberculosis exposure.

Her past medical history was unremarkable, and her menstrual history was normal. There was no known family history of autoimmune disease or malignancy. She had intermittently taken over-the-counter aceclofenac (NSAID) for joint pain and had no history of prior blood transfusions.

On examination, the patient appeared as a thin adolescent female weighing approximately 44 kilograms, with a height of 4 feet 8 inches (BMI ~21 kg/m²), alert and oriented. Vital signs were within normal limits. She appeared pallid and demonstrated mild, 1+ pitting edema of both lower legs. Multiple lymph nodes were palpable in the postauricular, anterior, and posterior cervical, supraclavicular, axillary, and inguinal regions. These nodes were generally non-tender, rubbery in consistency, and freely mobile, with the largest measuring approximately 3 by 4 centimeters.

Scalp inspection revealed marked hair loss in the frontal region without evidence of scarring, atrophy, or discoid lesions. Cutaneous examination demonstrated multiple non-palpable, non-blanching purpuric lesions confined to the lower extremities, as shown in Figure 1. There were no malar or discoid facial lesions, and the oral mucosa was unremarkable at the time of examination, though the patient reported a transient ulcer in the preceding weeks (single, on the buccal mucosa, not palate). Musculoskeletal evaluation revealed mild swelling and tenderness at the metacarpophalangeal and proximal interphalangeal joints, with full range of motion preserved. Cardiovascular, respiratory, abdominal, and neurological examinations were unremarkable.

Laboratory investigations were performed to assess hematologic, renal, and infectious causes (Table 1). The results revealed bicytopenia with pancytopenia features and mild thrombocytopenia, which, along with the purpuric rash, raised initial concern for hematologic disorders such as leukemia or lymphoma, infectious etiologies including tuberculosis and viral infections, and autoimmune processes.

**Table 1:** Hematologic and diagnostic workup RBC: red blood cell

Test	Result	Reference Range
Hemoglobin	8.4 g/dL	12–16 g/dL
White blood cell count	3,100 /mm³	4,500–11,000 /mm³
Platelets	100,000 /mm³	150,000–450,000 /mm³
Erythrocyte sedimentation rate (first hour)	81 mm	0–20 mm
Hematocrit	24.6%	36–46%
Peripheral smear	Pancytopenia, no hemolysis	–
Coombs test	Negative	Negative
Serum creatinine	0.9 mg/dL	0.6–1.1 mg/dL
Urinalysis	Trace protein, no RBC/casts	–
Chest X-ray	Normal	–
Abdominal ultrasound	Normal	–

Extended serology testing performed as finances allowed revealed a negative ANA by ELISA (IF not performed due to resource limitations), positive anti-dsDNA, and negative rheumatoid factor/anti-cyclic citrullinated peptide antibody (RF/anti-CCP) (Table 2). These immunologic findings, combined with the patient’s clinical features of alopecia, oral ulcer, inflammatory arthritis, and hematologic involvement, fulfilled four ACR classification criteria [15], supporting a diagnosis of SLE despite a negative ANA screen.

**Table 2:** Immunologic test results ANA (ELISA): antinuclear antibody (enzyme-linked immunosorbent assay); Anti-dsDNA: anti-double-stranded DNA antibody; RF/anti-CCP: rheumatoid factor/anti–cyclic citrullinated peptide antibody

Test	Result	Reference Range
ANA (ELISA)	Negative (OD 0.141; cutoff 0.150)	Negative
Anti-dsDNA (ELISA)	Positive (OD 3.537; cutoff 0.163)	Negative
RF/anti-CCP	Negative	Negative

An excisional lymph node biopsy was discussed to exclude lymphoma, Kikuchi-Fujimoto disease, tuberculosis, or other lymphoproliferative disorders, and to seek histopathologic confirmation of lupus lymphadenitis. However, the procedure was deferred due to financial limitations and family preference. A skin biopsy for the purpuric lesions was also considered, but not performed.

After multidisciplinary discussion, a working diagnosis of ANA-negative SLE was made. The patient met four ACR classification features-mucocutaneous, musculoskeletal, hematologic, and immunologic-consistent with SLE despite seronegative ANA testing [15].

Differential exclusion reasoning

Tuberculosis

The patient had no respiratory symptoms, cough, shortness of breath, or weight loss. She was fully immunized. A recent GeneXpert report (Cepheid, Sunnyvale, CA; done externally) was negative, and her chest X-ray and abdominal USG were normal. Due to financial constraints and lack of clinical suspicion, no further tuberculosis workup was performed.

Lymphoma/Kikuchi-Fujimoto Disease

While LAD was generalized, no prior USG, fine-needle aspiration cytology (FNAC), or excisional biopsy had been performed. Biopsy was discussed but deferred due to financial limitations and family preference. The patient’s clinical improvement with corticosteroid and hydroxychloroquine therapy supported an autoimmune etiology rather than malignancy.

Viral Infections

No history of recent viral illness or systemic symptoms was reported. Routine hematology, peripheral smear, and clinical course were not consistent with acute viral lymphadenitis.

Aplastic Anemia/Primary Bone Marrow Disorders

A bone marrow biopsy was not performed due to financial limitations. However, peripheral smear and reticulocyte count were reassuring, and cytopenias improved promptly with corticosteroids and transfusion, making primary bone marrow failure unlikely.

After multidisciplinary discussion, a working diagnosis of ANA-negative SLE was made, fulfilling mucocutaneous, musculoskeletal, hematologic, and immunologic ACR criteria [15] despite seronegative ANA testing (ELISA). 

Treatment and hospital course

The patient was admitted for close monitoring and initiation of therapy. Treatment consisted of oral prednisolone at 30 mg daily, with a planned taper after four weeks depending on clinical response, and hydroxychloroquine 200 mg daily. Supportive care included transfusion of two units of whole blood for symptomatic anemia and cytopenias, as well as paracetamol for symptomatic analgesia. During the initial immunosuppressive period, empiric prophylactic antibiotics, antivirals, and antifungals were administered per consulting team recommendations to reduce infectious risk. The patient received counseling regarding neutropenic precautions, including dietary and hygiene measures to minimize infection risk.

No high-dose intravenous methylprednisolone or cytotoxic agents were required, as there was no evidence of organ-threatening disease. Renal function remained within normal limits, urinalysis revealed only trace proteinuria without hematuria or casts, and neurologic examination remained unremarkable.

Response to treatment

Within two days of initiating corticosteroid and hydroxychloroquine therapy, the patient’s intermittent fevers resolved, and the purpuric lesions on the lower extremities (Figure 1) began to fade. Over the following one to two weeks, objective improvement was observed in lymph node size. Laboratory follow-up showed a trend toward normalization of blood counts, particularly hemoglobin and white blood cell levels, while platelet counts increased above admission values but remained mildly below the normal range. Joint pain and swelling improved markedly, with restored range of motion and decreased tenderness in the affected metacarpophalangeal and proximal interphalangeal joints. The transfused units of whole blood appeared temporally associated with transient improvement in hematologic parameters and reduction of purpuric lesions.

Follow-up

At a two-week outpatient review, the patient reported resolution of constitutional symptoms, including fatigue and fever. Blood counts showed further improvement compared to admission, though platelet counts remained slightly below the normal range (Table 1). The purpuric rash recurred mildly on the lower limbs but was less pronounced than prior to therapy (Figure 1). The family noted mild facial puffiness, consistent with corticosteroid administration.

The ongoing management plan included continuation of hydroxychloroquine 200 mg daily and completion of the steroid taper, with gradual dose reduction guided by clinical response and hematologic indices. The patient was advised to maintain strict follow-up in both the medicine and rheumatology clinics and to seek urgent evaluation for new-onset fever, bleeding manifestations, or neurologic or renal symptoms. Escalation of immunosuppressive therapy or reconsideration of lymph node or skin biopsy would be reserved for relapse or development of organ-threatening involvement.

Consent and ethical considerations

Written informed consent was obtained from the patient’s father for treatment, and verbal consent was obtained for publication of this case report and accompanying non-identifiable images. The treating team confirmed that no identifying information appears in the manuscript.

## Discussion

SLE is a chronic, multi-system autoimmune disease characterized by heterogeneous clinical manifestations and the production of autoantibodies, most notably ANA [1-6]. SLE predominantly affects females, and pediatric-onset cases represent a smaller but clinically significant subset, often presenting with more aggressive disease features compared to adult-onset SLE [1-4,6]. Despite advances in classification and diagnostics, atypical presentations, particularly ANA-negative disease, continue to pose significant diagnostic challenges [5,7,8]. ANA positivity is considered a hallmark of SLE, present in over 95% of cases, while ANA-negative SLE remains exceedingly rare, reported in fewer than 2%-5% of patients [7-10,12-14, 17]. Even in ANA-positive cases, unusual manifestations, such as acute pancreatitis, have been described, highlighting the broad spectrum of atypical presentations [14,18,19].

Generalized LAD is an uncommon initial manifestation of SLE. Historical cohorts reported LAD in up to 50% of patients, whereas more recent studies estimate a lower prevalence, and LAD rarely represents the dominant or first clinical feature [2,3,9,10]. In our patient, diffuse, painless lymph node enlargement preceded other classic lupus manifestations, creating a diagnostic dilemma and prompting consideration of infectious, malignant, and other autoimmune etiologies [11-14]. Similar cases in the literature demonstrate how extensive LAD and ANA negativity can mask the underlying diagnosis, often delaying recognition of SLE [12-14,17].

The differential diagnosis of generalized LAD is broad and includes infectious causes such as tuberculosis, Epstein-Barr virus, and HIV; hematologic malignancies; and autoimmune conditions such as Kikuchi-Fujimoto disease or Castleman’s disease [11-14,19]. Kikuchi-Fujimoto disease, in particular, has been frequently associated with SLE, either as a prodromal feature or as a coexisting condition, which can further complicate the diagnostic process [12-14]. This underscores the importance of maintaining a high index of suspicion for SLE when evaluating young patients with unexplained LAD, particularly females in high-risk populations [1-4,6].

ANA-negative SLE adds another layer of complexity. Previously considered an artifact of insensitive assays, ANA-negative lupus is now well-documented and often presents with other serological or clinical abnormalities such as anti-dsDNA antibodies, cytopenias, nephritis, or systemic inflammation [7-10,12-14,18,20-22]. Reports have described ANA-negative patients presenting with lupus nephritis, vasculitis, or other severe systemic involvement, emphasizing that ANA testing alone cannot exclude the diagnosis of SLE [12-14,18,20-22].

This case highlights three key teaching points. First, generalized LAD, although uncommon, can be an initial and sentinel feature of pediatric SLE and should be considered after ruling out infection and malignancy. Second, ANA negativity does not preclude the diagnosis of lupus, particularly when other immunologic or clinical findings support the disease. Third, early recognition is critical for initiating timely therapy, avoiding unnecessary invasive procedures, and preventing organ damage [7,12,17,22].

Our report adds to the limited but growing literature on ANA-negative SLE with generalized LAD, reinforcing the importance of clinical vigilance in atypical presentations. Future studies may clarify whether ANA-negative SLE represents a distinct immunopathologic subset with unique clinical trajectories [7,12].

## Conclusions

ANA-negative SLE is a rare but clinically important entity, especially in pediatric patients. Generalized LAD as an initial manifestation is uncommon and can mimic infectious, malignant, or other autoimmune conditions, creating diagnostic challenges. This case emphasizes that ANA negativity does not exclude SLE, and comprehensive clinical assessment alongside targeted immunologic testing is essential for accurate diagnosis and timely management. Clinicians should maintain a high index of suspicion in atypical presentations, as early recognition allows prompt therapy, reduces morbidity, and may prevent unnecessary invasive investigations. Reporting such rare cases contributes to understanding the diverse spectrum of SLE and may inform future research on potential immunopathologic subsets.

## Appendices

Appendix A 

**Table 3:** Classification criteria for systemic lupus erythematosus (SLE) Comparison of the American College of Rheumatology (ACR 1997), Systemic Lupus International Collaborating Clinics (SLICC 2012), and European League Against Rheumatism/American College of Rheumatology (EULAR/ACR 2019) classification criteria for SLE. The table summarizes the key components and diagnostic thresholds across criteria used to classify SLE in clinical and research settings. Sources: [15,16,23]

Criteria Set	Criteria/Components	Notes/Threshold
ACR (1997)	1. Malar rash 2. Discoid rash 3. Photosensitivity 4. Oral ulcers 5. Arthritis (non-erosive) 6. Serositis (pleuritis/pericarditis) 7. Renal disorder (proteinuria or cellular casts) 8. Neurologic disorder (seizures/psychosis) 9. Hematologic disorder (hemolytic anemia, leukopenia, lymphopenia, thrombocytopenia) 10. Immunologic disorder (anti-dsDNA, anti-Sm, antiphospholipid) 11. ANA positivity	Diagnosis requires ≥4 of 11 criteria, either serially or simultaneously
SLICC (2012)	Clinical criteria: 1. Acute/subacute cutaneous lupus 2. Chronic cutaneous lupus 3. Oral/nasal ulcers 4. Nonscarring alopecia 5. Synovitis (≥2 joints) 6. Serositis 7. Renal involvement 8. Neurologic involvement 9. Hemolytic anemia 10. Leukopenia/lymphopenia 11.Thrombocytopenia Immunologic criteria: 1. ANA positivity 2. Anti-dsDNA 3. Anti-Sm 4. Antiphospholipid antibodies 5. Low complement (C3, C4) 6. Direct Coombs (if no hemolytic anemia)	Diagnosis requires ≥4 criteria, with at least 1 clinical and 1 immunologic OR biopsy-proven lupus nephritis with positive ANA or anti-dsDNA
EULAR/ACR (2019)	Entry criterion: 1. ANA ≥1:80 by immunofluorescence. Additive criteria (weighted points): 1. Constitutional: Fever (2 pts) 2. Hematologic: Leukopenia (3 pts), Thrombocytopenia (4 pts), Autoimmune hemolysis (4 pts) 3. Neurologic: Delirium (2 pts), Psychosis (3 pts), Seizure (5 pts) 4. Mucocutaneous: Non-scarring alopecia (2 pts), Oral ulcers (2 pts), Subacute cutaneous (4 pts), Acute cutaneous (6 pts) 5. Serosal: Pleural/pericardial effusion (5 pts), Acute pericarditis (6 pts). 6. Musculoskeletal: Joint involvement (6 pts) 7. Renal: Proteinuria >0.5 g/24h (4 pts), Biopsy Class II/V (8 pts), Biopsy Class III/IV (10 pts) 8. Immunologic: Anti-dsDNA (6 pts), Anti-Sm (6 pts), Low C3/C4 (3 pts)	Total ≥10 points required after meeting entry criterion (ANA positivity)
